# Retraction Note: New molecular tools in *Neospora caninum* for studying apicomplexan parasite proteins

**DOI:** 10.1038/s41598-018-28052-2

**Published:** 2018-07-13

**Authors:** Caroline M. Mota, Allan L. Chen, Kevin Wang, Santhosh Nadipuram, Ajay A. Vashisht, James A. Wohlschlegel, Tiago W. P. Mineo, Peter J. Bradley

**Affiliations:** 1 Laboratory of Immunoparasitology “Dr. Mário Endsfeldz Camargo”, Institute of Biomedical Sciences, Universidade Federal de Uberlândia, Uberlândia, Minas Gerais Brazil; 2 Department of Microbiology, Immunology and Molecular Genetics, University of California, Los Angeles, Los Angeles, California USA; 3 Department of Biological Chemistry and Institute of Genomics and Proteomics, University of California, Los Angeles, Los Angeles, California USA; 4 Molecular Biology Institute, University of California, Los Angeles, Los Angeles, California USA

Retraction of: *Scientific Reports* 10.1038/s41598-017-03978-1, published online 19 June 2017

The authors are retracting this Article.

In the follow-up experiments on this work, we discovered that the strain of *Neospora caninum* had been inadvertently switched with an avirulent ∆*rop54* strain of *Toxoplasma gondii*. As such, the results presented throughout the manuscript are in the incorrect parasite background. The conclusions regarding the utility of this system in *Neospora* cannot be supported until these experiments are repeated in *N. caninum*.

All authors agree to the retraction of the Article.

